# MRI-based pseudo-CT sequences as a radiation-free alternative to CT for obstetric pelvimetry: a proof-of-concept study

**DOI:** 10.1186/s41747-025-00585-y

**Published:** 2025-05-19

**Authors:** Caroline Chabot, Mathilde Haegeman, Eya Chaouch, Dana Dumitriu, Renaud Menten, Patricia Steenhaut, Pierre Bernard, Perrine Triqueneaux, Nicolas Michoux, Frédéric E. Lecouvet

**Affiliations:** 1 Department of Medical Imaging, Institut de Recherche Expérimentale et Clinique (IREC), Institut du Cancer Roi Albert II, Cliniques Universitaires Saint-Luc, Université Catholique de Louvain (UCL), Brussels, Belgium; 2 Department of Obstetrics, Cliniques Universitaires Saint-Luc, Université Catholique de Louvain (UCL), Brussels, Belgium

**Keywords:** Magnetic resonance imaging, Obstetrics, Pelvimetry, Reproducibility of results, Tomography (x-ray computed)

## Abstract

**Background:**

Pelvimetry is essential in obstetrics for delivery planning. While computed tomography (CT) is the standard, magnetic resonance imaging (MRI) offers a radiation-free alternative with zero echo time (ZTE) and black bone (BB) sequences providing high bone-to-soft tissue contrast within short scan times. This proof-of-concept study evaluates the reliability of these sequences and the agreement with CT for pelvimetry in a predominantly elderly population.

**Methods:**

This retrospective study included 21 female patients who underwent 3-T whole-body MRI including ZTE and BB sequences and ^18^fluorodeoxyglucose (^18^F-FDG) positron emission tomography (PET)/CT with optimized low-dose whole-body CT. Obstetric conjugate diameter (OCD), interspinous diameter (ISD), and median transverse diameter (MTD) were measured by five radiologists. Intra-reader, inter-reader, and inter-technique agreement were assessed using intraclass correlation coefficient (ICC) and repeatability/reproducibility coefficients.

**Results:**

Intra-reader agreement was good regardless of diameter or reader: all ICC ≥ 0.90, repeatability ranging from ± 0.26 to ± 0.48 cm (CT), ± 0.30 to ± 0.52 cm (BB), and ± 0.29 to ± 0.67 cm (ZTE). The inter-reader agreement was good regardless of sequence: all ICC ≥ 0.88, reproducibility ranging from ± 0.39 to ± 0.42 (OCD), ± 0.26 to ± 0.51 cm (ISD), and ± 0.53 to ± 0.58 cm (MTD). ZTE and BB showed similar agreement with CT: ± 0.57 to ± 0.81 cm when including inter-reader variability; ± 0.34 to ± 0.47 cm for only intra-reader variability.

**Conclusion:**

ZTE and BB sequences provided reliable measurements with good agreement with CT, for obstetric pelvimetry. Further validation in the context of pregnancy is needed.

**Relevance statement:**

MRI-based pseudo-CT sequences are a promising radiation-free alternative to CT for obstetric pelvimetry, offering the prospect of accurate, reliable measurements of pelvic diameters in pregnant women.

**Trial registration:**

The population included female patients with suspected multiple myeloma from a previous prospective oncology trial (ClinicalTrials.gov: NCT05381077).

**Key Points:**

- This study explores pseudo-CT MRI sequences for radiation-free non-invasive obstetric pelvimetry.
- Pseudo-CT zero echo time and black bone sequences provide repeatable and reproducible measurements of pelvic diameters.
- Pseudo-CT MRI sequences show good inter-technique agreement with the reference CT.

**Graphical Abstract:**

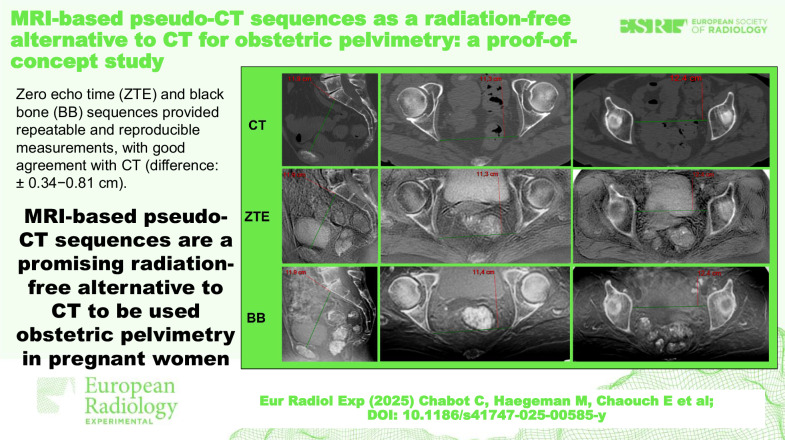

## Background

Imaging-based pelvimetry has been widely used to assess the diameter of the mother’s pelvis to help the obstetrician decide between cesarean section and vaginal delivery. Pelvimetry can be used postpartum, especially after complications such as prolonged labor or previous cesarean section, or antepartum to assess the mode of delivery at term. The main indications are breech presentation or suspected cephalopelvic disproportion between fetal and maternal pelvic dimensions [1, 2]. Radiographs have been used for this purpose since the 1940s, although computed tomography (CT) pelvimetry, introduced in the 1980s, quickly became the preferred method due to advantages such as simplified procedure, reduced fetal radiation, and multiplanar measurements [3–6]. Reference values for key pelvic diameters have been established, and the CT technique has been optimized to reduce radiation exposure with advances in multidetector and three-dimensional (3D) imaging [7–9].

In parallel, magnetic resonance imaging (MRI) pelvimetry emerged as a radiation-free alternative, initially using sagittal and transverse T1- or T2-weighted sequences. Gradual improvements, including the transition from spin-echo to gradient-echo sequences, improved cortical definition and enabled 3D reconstructions [10–16]. Studies have also documented reference measurements and variability, positioning MRI as the preferred imaging modality over CT and radiographs for pelvimetry [14, 17–19]. However, MRI pelvimetry is not routinely used due to issues such as MRI availability, higher costs, and longer scan times, which can be challenging for pregnant women who need to remain still [19].

Recent MRI innovations, particularly pseudo-CT sequences, allow for high bone-to-soft tissue contrast in reasonable scan times. Zero echo time (ZTE) and black bone (BB) sequences use very short echo times (1 ms or less for ZTE) to capture signals from rapidly decaying short-T2 tissue, making bone structures stand out. In addition, BB imaging uses a 3D T1-weighted spoiled gradient-echo sequence, which renders bone structures dark against a high-contrast background and has shown potential for various musculoskeletal applications, including degenerative joint disease, fractures, and tumors [20–24]. Pseudo-CT synthetic sequences derived from ZTE images have recently been investigated for the measurement of pelvic and hip dimensions in non-obstetric contexts, with promising results [25].

The aim of the present study was to assess the value of native ZTE and BB pseudo-CT sequences in providing reliable pelvic diameter measurements in obstetrics. This retrospective study used MRI acquisitions from a previous prospective oncology study to evaluate (i) the repeatability and reproducibility of pelvimetric measurements from ZTE and BB images and (ii) their agreement with reference CT measurements. It was designed to determine the feasibility of using these sequences as a substitute for CT before a prospective trial is undertaken in pregnant women.

## Methods

### Patient population

**Fig. 1:**
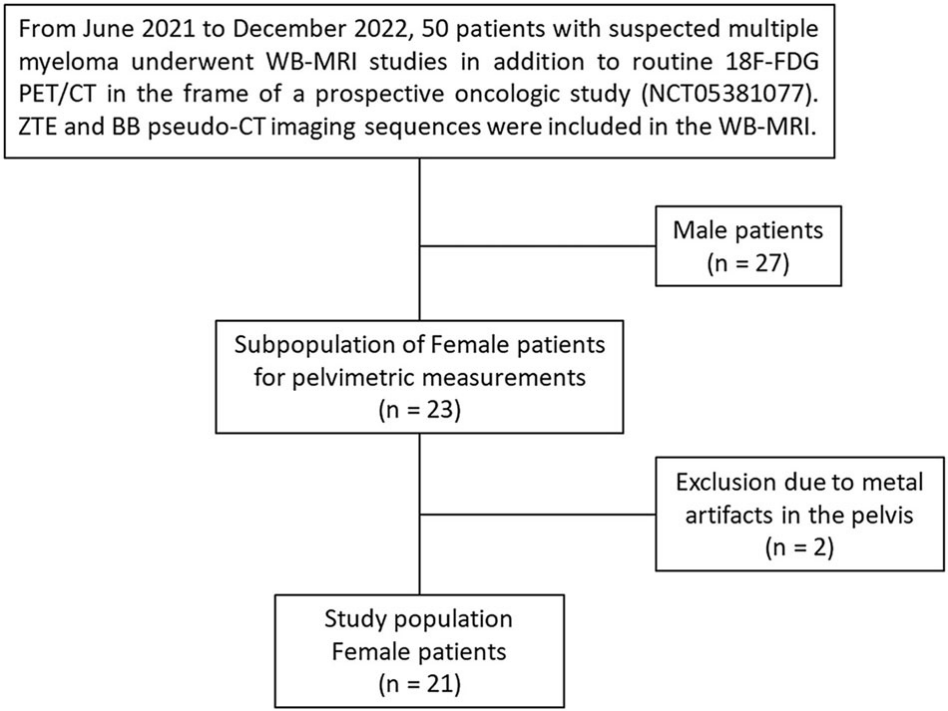
Flowchart shows participant inclusion and exclusion. *18F-FDG PET/CT*, ^18^F-Fluorodeoxyglucose positron emission tomography/computed tomography; BB, Black bone sequence; CT, Computed tomography; WB-MRI, Whole-body magnetic resonance imaging; ZTE, Zero Echo time sequence

This retrospective study was approved by the institutional ethics committee (2023/07AVR/174). The population included female patients with suspected multiple myeloma from a previous oncology trial (ClinicalTrials.gov: NCT05381077) who, after approval by the ethics committee (2020/27JUL/380), had undergone whole-body MRI with pseudo-CT sequences and optimized CT in positron emission tomography (PET)/CT scans for skeletal screening (Fig. 1) [26]. Exclusion criteria were incomplete whole-body MRI or pseudo-CT sequences and metallic artefacts due to orthopedic hardware in the pelvis.

### MRI protocol

**Fig. 2:**
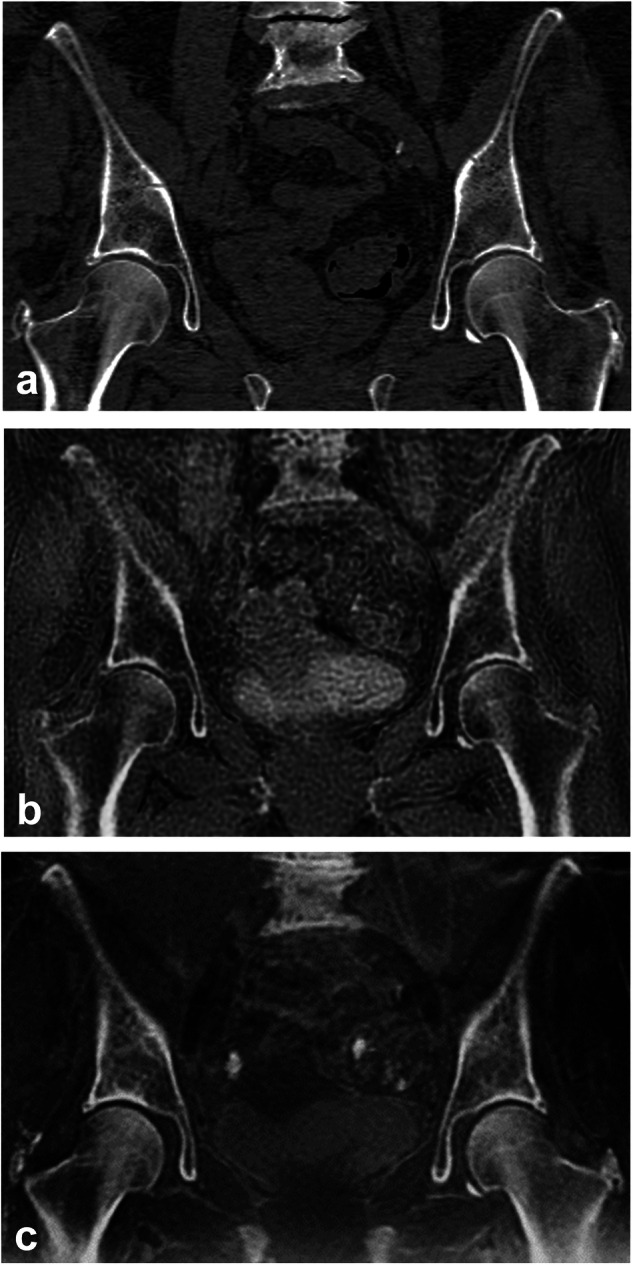
A 67-year-old woman with multiple myeloma. Coronal reformatted images of the pelvis illustrate the different available examinations and sequences: (**a**) reference computed tomography; (**b**) inverted gray-scale zero echo time MRI sequence; and (**c**) inverted gray-scale black bone MRI sequence. MRI, Magnetic resonance imaging

All MRI examinations were performed on a 3-T scanner (Signa Premier, GE Healthcare, WI, USA). The protocol covered the body from vertex to mid-thigh and included a 3D T1-weighted fast spin-echo sequence, diffusion-weighted imaging with apparent diffusion coefficient mapping, and a Dixon-based 3D T1-weighted gradient-echo sequence, producing in-phase, out-of-phase, fat-only, water-only, and fat fraction maps [26, 27]. Two pseudo-CT sequences, ZTE and BB, were added, focusing on the lumbar spine, pelvis, and proximal femurs, where skeletal involvement by myeloma is common [26–28]. A detailed explanation of these sequences can be found in the literature [29, 30]. The ZTE sequence uses continuous readout gradients, allowing an echo time of 0 ms to enhance cortical and trabecular bone contrast. The BB sequence, a 3D spoiled gradient-echo sequence with a short echo time, visualizes mineral bone as black; the Dixon analysis was used to study the bone marrow on water-only and fat-only images. Both sequences use a low flip angle to minimize contrast within the soft tissues and improve bone delineation. ZTE and BB sequences were displayed with gray-scale inversion to match the bone appearance on CT (Fig. 2). Acquisition time was 2:50 min:s for the ZTE sequence, 2:16 min:s for the BB sequence. MRI technical parameters are detailed in Table 1.

**Table 1:** Acquisition parameters of the pseudo-CT MRI sequences

Sequence	Zero echo time	Black bone
Acquisition mode and plane	3D coronal	3D axial
Field of view (cm^3^)	44 × 44 × 29	50 × 40 × 26.5
Voxel size (mm^3^)	1.6 × 1.6 × 1.6	1.6 × 1.6 × 1.6
Repetition time/Echo time (ms)	−/0	4.2/1.1, 2.4
Flip angle (°)	1	1
Number of excitations	2	2
Breathing	Free	Free
Time of acquisition per stack (min:s)	2:50	2:16
Number of stacks	1	1

The parameters of the sequences used for bone marrow study in the frame of the “mother” trial in myeloma patients (anatomical T1-weighted sequences, functional diffusion-weighted sequences with apparent diffusion coefficient measurements, and Dixon-based sequences with fat fraction quantification) have been reported elsewhere [26]. *3D* Three-dimensional, *CT* Computed tomography, *MRI* Magnetic resonance imaging

### Optimized CT examination

CT images were part of a PET/CT protocol optimized for bone evaluation, acquired on a Philips Vereos scanner (Philips, Best, The Netherlands). CT parameters included 120 kVp, current modulation, and 64-slice collimation with 0.625-mm pitch. A 1-mm voxel matrix with a Y-sharp filter for bone was used for reconstruction. Patients were positioned supine with arms along the trunk to match the MRI positioning. To ensure the same positioning, MRI and nuclear medicine technologists were trained together before the study. Detailed image parameters can be found in a previous paper [26]. The acquisition time of the CT for covering the pelvic area was 10 s.

### MRI image analysis

**Fig. 3:**
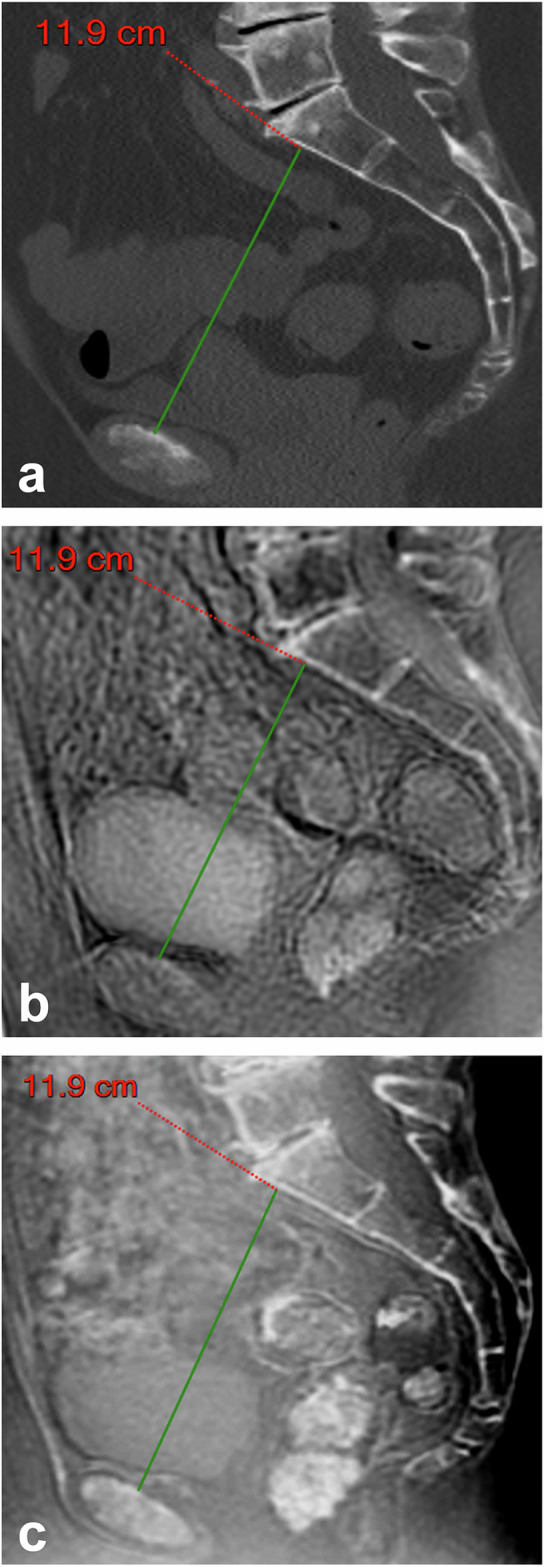
A 67-year-old woman with multiple myeloma, sagittal reformatted images for measuring the obstetric conjugate diameter (green line): (**a**) reference computed tomography; (**b**) inverted gray-scale zero echo time MRI sequence; and (**c**) inverted gray-scale black bone MRI sequence. MRI, Magnetic resonance imaging

**Fig. 4:**
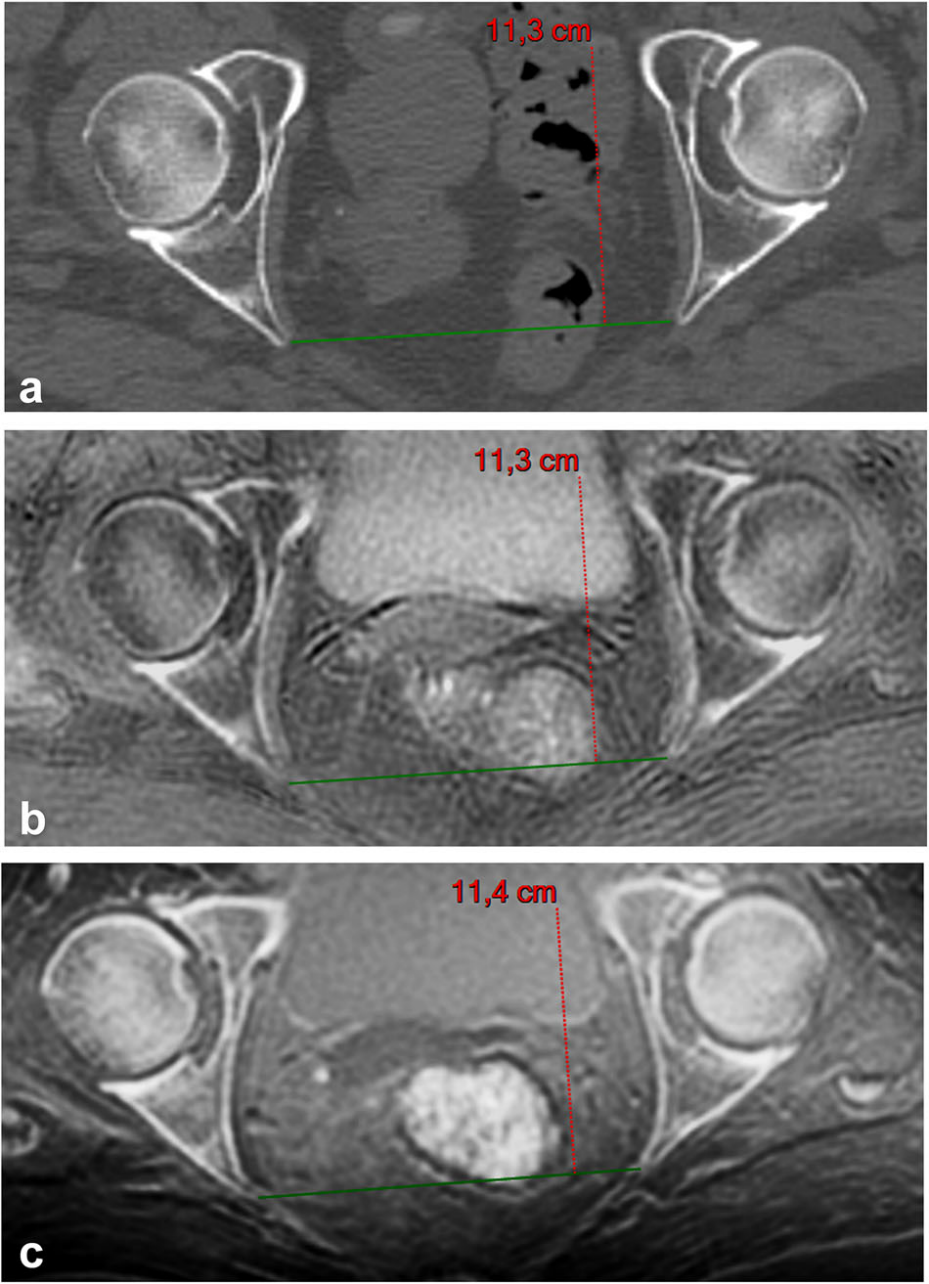
A 67-year-old woman with multiple myeloma, axial reformatted images for measuring the interspinous distance (green line): (**a**) reference computed tomography; (**b**) inverted gray-scale zero echo time MRI sequence; and (**c**) inverted gray-scale black bone MRI sequence. MRI, Magnetic resonance imaging

**Fig. 5:**
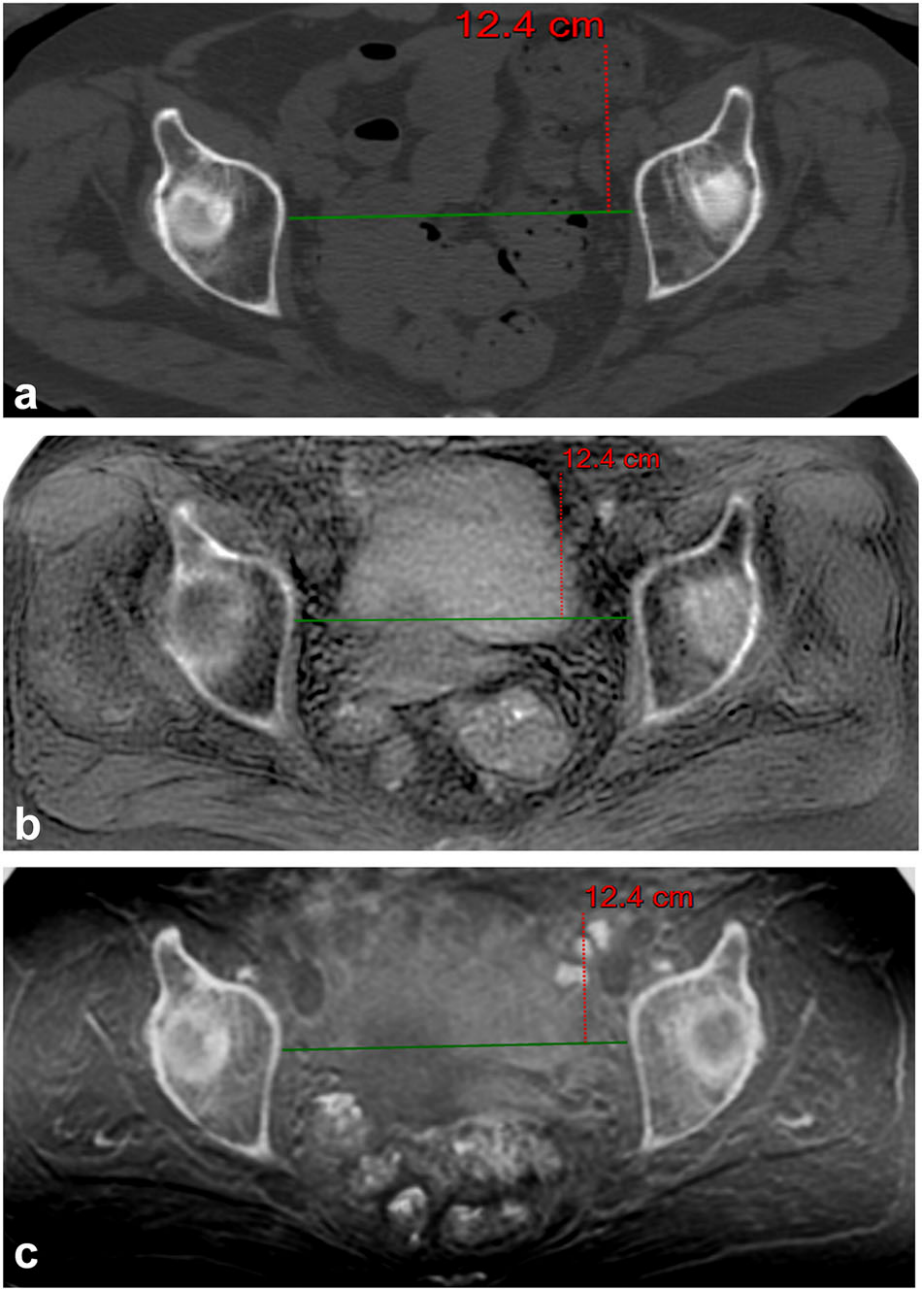
A 67-year-old woman with multiple myeloma, axial reformatted images for measuring the median transverse diameter (green line): (**a**) reference computed tomography; (**b**) Inverted gray-scale zero echo time MRI sequence; and (**c**) inverted gray-scale black bone MRI sequence. MRI*,* Magnetic resonance imaging

Five readers analyzed all images on PACS workstations (Carestream Vue, Philips, Best, The Netherlands): two pediatric radiologists with 20 and 25 years of experience in pelvimetry (Reader 1. R.M.; Reader 2, D.D.) and three residents (Reader 3, M.H., 5th-year resident; Reader 4, C.C., 3rd-year resident; Reader 5, E.C., 5th-year resident) with different levels of training in pelvimetric measurements. They independently performed the three standard measurements required by obstetricians: the obstetric conjugate diameter (OCD), the interspinous diameter (ISD), and the median transverse diameter (MTD) (Figs. 3, 4, and 5).

The imaging planes for these measurements were defined as follows:

- OCD, measured in the sagittal plane from the top of the promontory to 1 cm below the pubic symphysis;
- MTD, measured in the axial plane perpendicular to the OCD, parallel to the line passing through the femoral heads; and
- ISD, measured in the axial plane at the level of the smallest diameter between the ischiatic spines [31].

Measurements were performed on ZTE, BB, and CT images in a blinded, randomized fashion. MRI sequences were read after inverting the gray scale to highlight cortical and trabecular bone as “white structures”, similar to the CT. Both MRI and CT were analyzed using the Picture Archiving and Communication System windowing, multiplanar reformation, and scrolling tools. Two junior readers (Reader 3 and Reader 4) repeated the measurements at 2-month intervals to avoid recall bias.

### Statistical analysis

Measurements are presented as means (or medians when the data distributions were non-normal according to Shapiro-Wilk’s test) with their 95% confidence intervals (CIs).

Intra-reader agreement was assessed from the first and second readings from Reader 3 and Reader 4 by using a “two-way random effects” intraclass correlation coefficient (ICC) [32]. Similarly, inter-reader agreement was assessed from the first reading of all readers. The strength of agreement was interpreted as follows: ICC < 0.20, poor; 0.20 ≤ ICC < 0.40, fair; 0.40 ≤ ICC < 0.60, moderate; 0.60 ≤ ICC < 0.80, good; ICC ≥ 0.80, very good. These analyses were performed for each of the three pelvic diameters. Intra-reader agreement was also assessed in terms of mean bias (in cm) and in terms of repeatability coefficient (in cm) estimated at a 95% confidence level using the Bland-Altman approach [33]. Inter-reader agreement was assessed as the coefficient of reproducibility (in cm) from the first reading from “All readers”. Inter-technique agreement was assessed in terms of mean bias (in cm) and in terms of coefficient of agreement (in cm) for “All readers” from the first reading, and for Reader 3 and Reader 4 from their two readings. A Bland-Altman approach with repeated measurements (*n* = 5 measures in cases of “All readers” and *n* = 2 measures in case of Reader 3 and Reader 4) was performed for this purpose. These analyses were performed for each of the three pelvic diameters. A one-sample, two-sided *t*-test was then performed with the null hypothesis that the mean bias was equal to 0. In the repeatability analysis, as three techniques and two readers were included for each pelvic diameter, a Bonferroni correction was applied, and a *p*-value < 0.0083 was considered statistically significant. In the inter-technique analysis, as two comparisons (CT *versus* BB and CT *versus* ZTE) and three sets of measurements (“All readers”, Reader 3 and Reader 4) were included for each pelvic diameter, a *p*-value < 0.0083 was also considered as significant. All calculations were performed by N.M. using Medcalc v23.0.5 software.

## Results

### Patient characteristics

Out of 23 female patient files, including all imaging sequences, 21 were included in the study, and 2 were excluded due to the presence of metallic artifacts induced by prosthetic material (Fig. 1). The patients had a mean age of 66 years (range, 60–75; median, 66 years). The indication for PET/CT and whole-body MRI studies was the work-up of suspected myeloma in all cases.

### Pelvimetric measurements

Mean measurements of the obstetric diameters by the five readers are given in Table 2 (Figs. 3, 4, and 5).

**Table 2:** Pelvic diameters by computed tomography and MRI

Reader			OCD	ISD	MTD
Computed tomography					
R1			11.2 (10.7; 11.7)	10.6 (10.1; 11.0)	12.6 (12.2; 12.9)
R2			11.3 (10.8; 11.7)	10.6 (10.2; 11.1)	12.9 (12.5; 13.4)
R3			11.2 (10.7; 11.7)	10.8 (10.4; 11.2)*	12.7 (12.3; 13.1)
R4			11.3 (10.8; 11.8)	10.8 (10.3; 11.2)	12.9 (12.6; 13.3)
R5			11.4 (10.9; 11.9)	10.8 (10.3; 11.3)*	12.8 (12.4; 13.1)
Zero echo time MRI					
R1			11.2 (10.7; 11.6)	11.2 (10.1; 11.4)*	12.6 (12.2; 13.0)
R2			11.4 (11.0; 11.9)	11.3 (10.5; 11.8)*	13.1 (12.6; 13.5)
R3			11.3 (10.9; 11.8)	11.0 (10.3; 11.4)*	12.7 (12.3; 13.1)
R4			11.4 (10.9; 11.9)	10.9 (10.3; 11.5)*	12.9 (12.6; 13.3)
R5			11.3 (10.9; 11.8)	10.9 (10.4; 11.4)	12.8 (12.4; 13.2)
Black bone MRI					
R1			11.2 (10.7; 11.6)	11.2 (10.1; 11.3)*	12.6 (12.2; 13.0)
R2			11.4 (11.0; 11.9)	11.3 (10.3; 11.7)*	13.0 (12.5; 13.4)
R3			11.3 (10.8; 11.8)	10.9 (10.2; 11.2)*	12.7 (12.3; 13.1)
R4			11.4 (10.9; 11.9)	10.8 (11.3; 11.3)	12.9 (12.6; 13.3)
R5			11.4 (10.9; 11.9)	10.9 (10.3; 11.5)	12.7 (12.4; 13.1)

Values are means with the exception of those marked with *, which are medians. Readers 3 and 4 performed measurements twice

*ISD* Interspinous diameter, *MRI* Magnetic resonance imaging, *MTD* Median transverse diameter, *OCD* Obstetric conjugate diameter

### Intra-reader agreement for pelvic diameter measurements

ICC, mean bias and repeatability coefficients are provided in Table 3. Repeatability was good regardless of the imaging technique, diameter and reader (all ICC values ≥ 0.90). In CT, considering Reader 3 and Reader 4, the repeatability coefficient ranged from ± 0.26 cm when measuring OCD to ± 0.48 cm when measuring MTD. In MRI, considering Reader 3 and Reader 4, the repeatability coefficient ranged from ± 0.29 cm when measuring OCD to ±  0.67 cm when measuring MTD.

**Table 3:** Intra- and inter-reader agreement on the measurements of the three pelvic diameters

	Intra-reader agreement				Inter-reader agreement		
	Reader	Mean bias	Repeatability	ICC	Readers	Reproducibility	ICC
Obstetric conjugate diameter							
Computed tomography	R3	-0.02 (-0.08; +0.04)	0.26 (0.22; 0.30)	0.99 (0.98; 1.00)	All	0.39 (0.27; 0.51)	0.97 (0.94; 0.98)
	R4	+0.07 (-0.04; +0.17)	0.44 (0.37; 0.51)	0.98 (0.94; 0.99)			
Black bone MRI	R3	-0.01 (-0.08; +0.06)	0.30 (0.26; 0.35)	0.99 (0.98; 1.00)	All	0.42 (0.29; 0.55)	0.96 (0.92; 0.98)
	R4	+0.01 (-0.10; +0.12)	0.47 (0.40; 0.55)	0.97 (0.94; 0.99)			
Zero echo time MRI	R3	-0.02 (-0.09; +0.04)	0.29 (0.24; 0.34)	0.99 (0.97; 1.00)	All	0.41 (0.29; 0.54)	0.96 (0.92; 0.98)
	R4	+0.09 (-0.03; +0.21)	0.52 (0.44; 0.60)	0.96 (0.91; 0.99)			
Interspinous diameter							
Computed tomography	R3	-0.10 (-0.17; -0.04)^1^	0.27 (0.23; 0.32)	0.99 (0.93; 1.00)	All	0.26 (0.18; 0.35)	0.98 (0.96; 0.99)
	R4	+0.18 (+ 0.08; +0.27)^2^	0.40 (0.34; 0.46)	0.97 (0.81; 0.99)			
Black bone MRI	R3	-0.07 (-0.15; +0.01)	0.35 (0.29; 0.40)	0.98 (0.95; 0.99)	All	0.51 (0.35; 0.66)	0.94 (0.88; 0.97)
	R4	+0.24 (+ 0.12; +0.36)^3^	0.52 (0.44; 0.60)	0.93 (0.63; 0.98)			
Zero echo time MRI	R3	-0.03 (-0.10; +0.05)	0.33 (0.28; 0.38)	0.99 (0.97; 0.99)	All	0.42 (0.29; 0.55)	0.96 (0.91; 0.98)
	R4	+0.05 (-0.11; +0.20)	0.67 (0.56; 0.78)	0.94 (0.87; 0.98)			
Median transverse diameter							
Computed tomography	R3	-0.09 (-0.15; -0.03)^4^	0.26 (0.22; 0.30)	0.98 (0.93; 0.99)	All	0.53 (0.37; 0.69)	0.90 (0.80; 0.96)
	R4	+0.28 (+ 0.17; +0.39)^5^	0.48 (0.40; 0.55)	0.90 (0.30; 0.97)			
Black bone MRI	R3	-0.16 (-0.26; -0.05)^6^	0.46 (0.39; 0.54)	0.94 (0.79; 0.98)	All	0.58 (0.40; 0.76)	0.88 (0.77; 0.94)
	R4	+0.24 (+ 0.13; +0.35)^7^	0.47 (0.40; 0.55)	0.92 (0.50; 0.97)			
Zero echo time MRI	R3	-0.11 (-0.19; -0.04)^8^	0.31 (0.26; 0.35)	0.97 (0.88; 0.99)	All	0.53 (0.36; 0.69)	0.91 (0.78; 0.96)
	R4	+0.23 (+ 0.13; +0.33)^9^	0.44 (0.37; 0.51)	0.93 (0.52; 0.98)			

ICC was used for assessing the intra-reader agreement in R3 and R4. Intra-reader agreement was also assessed as mean bias (in cm) and in terms of repeatability coefficient (in cm) estimated at a 95% confidence level. Inter-reader agreement was assessed in terms of reproducibility coefficient (in cm) following the same principle. The Table reads as follows. For computed tomography, when the Obstetric conjugate diameter was measured by R3 (upper left part of the table), the intra-reader agreement was very good (ICC = 0.99). According to the one-sample *t*-test, the mean bias (-0.02 cm) was not significantly different from 0. Measurements were repeatable at ± 0.26 cm. For computed tomography, when the OCD was measured by all readers (upper right part of the table), the inter-reader agreement was very good (ICC = 0.97). Measurements were reproducible at ± 0.39 cm. Statistical significance was declared at *p* < 0.0083. The following *t*-tests are significant: ^1^*p* = 0.0026, ^2^*p* = 0.0008, ^3^*p* = 0.0005, ^4^*p* = 0.0073, ^5^*p* < 0.0001, ^6^*p* = 0.0063, ^7^*p* = 0.0002, ^8^*p* = 0.0031, ^9^*p* = 0.0002

*ICC* Intraclass correlation coefficient, *MRI* Magnetic resonance imaging

When the mean bias was significantly different from 0, its worst value was 0.28 cm (95% CI 0.17–0.39 cm) when measuring MTD using CT.

### Inter-reader agreement for pelvic diameter measurements

ICC and reproducibility coefficients are shown in Table 3. Reproducibility was good regardless of the imaging technique and diameter (all ICC values ≥ 0.88). In CT, the reproducibility coefficient ranged from ± 0.26 cm for ISD to ± 0.53 cm for MTD. In MRI, the reproducibility coefficient ranged from ± 0.41 cm for OCD to ± 0.58 cm for MTD.

### Inter-technique agreement for diameter measurements

Mean bias and agreement coefficients are shown in Table 4. Across all readers and diameters, agreement between CT and MRI was found to be similar regardless of the MRI sequence. Agreement between CT and BB ranged from ± 0.57 cm to ± 0.78 cm, while agreement between CT and ZTE ranged from ± 0.59 cm to ± 0.81 cm. When individual readers (Reader 3 or Reader 4) were considered, *i.e*., when inter-reader variability was removed, but intra-reader variability was maintained, better inter-technique agreement was observed, regardless of the MRI sequence. The agreement between CT and BB thus ranged from ± 0.34 cm to ± 0.52 cm, while the agreement between CT and ZTE ranged from ± 0.41 cm to ± 0.53 cm. The 95% CIs associated with the agreement values did not overlap (except for the 95% CI associated with the comparison CT *versus* ZTE for Reader 4, which overlapped the 95% CI associated with the comparisons CT *versus* BB and CT *versus* ZTE in “All readers”), this difference in inter-technique agreement between “All readers” and individual readers was considered as significant at *p* < 0.05.

**Table 4:** Inter-technique agreement (with CT-derived values as a reference) for the measurements of the three pelvic diameters

	Reader	Mean bias	Agreement		Reader	Mean bias	Agreement
Obstetric conjugate diameter							
CT *versus* BB	All	-0.06 (-0.12; 0.00)	0.57 (0.53; 0.62)	CT *versus* ZTE	All	-0.05 (-0.14; 0.04)	0.63 (0.57; 0.69)
	R3	-0.08 (-0.16; 0.01)	0.41 (0.36; 0.47)		R3	-0.08 (-0.18; 0.02)	0.47 (0.40; 0.53)
	R4	-0.08 (-0.15; -0.00)	0.46 (0.41; 0.51)		R4	-0.04 (-0.11; 0.03)	0.47 (0.42; 0.52)
ISD interspinous diameter							
CT *versus* BB	All	-0.07 (-0.15; 0.01)	0.62 (0.56; 0.67)	CT *versus* ZTE	All	-0.16 (-0.25; -0.07)^1^	0.59 (0.53; 0.65)
	R3	0.05 (-0.01; 0.11)	0.34 (0.31; 0.38)		R3	-0.04 (-0.13; 0.04)	0.44 (0.38; 0.50)
	R4	0.08 (0.01; 0.14)	0.52 (0.47; 0.56)		R4	-0.03 (-0.12; 0.05)	0.56 (0.50; 0.62)
Median transverse diameter							
CT *versus* BB	All	-0.00 (-0.08; 0.08)	0.78 (0.73; 0.84)	CT *versus* ZTE	All	-0.03 (-0.14; 0.07)	0.81 (0.73; 0.88)
	R3	-0.00 (-0.06; 0.06)	0.40 (0.36; 0.44)		R3	0.00 (-0.07; 0.08)	0.41 (0.36; 0.46)
	R4	-0.03 (-0.07; 0.01)	0.52 (0.49; 0.54)		R4	-0.01 (-0.06; 0.05)	0.53 (0.49; 0.56)

The agreement was assessed in terms of mean bias (in cm) and in terms of reproducibility coefficient (in cm) with 95% confidence intervals in brackets. A Bland-Altman analysis with repeated measurements was applied (*n* = 5 measures in the case of “All readers” and *n* = 2 measures in the case of R3 and R4). Example of table interpretation (for OCD): according to the one-sample *t*-test, the mean bias (-0.06 cm) was not significantly different from 0; measures were found to be reproducible at ± 0.57 cm; stated differently, OCD measurements were equivalent to CT and to BB MRI at ± 0.57 cm. Statistical significance was declared at *p* < 0.0083. 95% confidence intervals are given in brackets. Mean bias was computed as the mean difference “CT minus MRI”. ^1^*p* = 0.0011

*BB* Black bone, *CT* Computed tomography, *OCD* Obstetric conjugate diameter, *ZTE* Zero echo time

Across all readers, it was also found that the inter-observer agreement for OCD and ISD was better than the inter-observer agreement for MTD, regardless of the MRI sequence (again, the 95% CIs associated with OCD/ISD on the one hand and MTD on the other hand did not overlap, *i.e*., the difference in inter-observer agreement was significant at *p* < 0.05). When considering individual readers (Reader 3 or Reader 4), no difference in inter-technique agreement was observed according to diameter.

Across all readers and diameters considered, when the inter-technique mean bias was significantly different from 0, its worst value was 0.16 cm (95% CI 0.07–0.25 cm). Across individual readers (Reader 3 or Reader 4), no inter-technique mean bias was observed, regardless of diameter.

## Discussion

A study published in 1998 showed that at that time MRI was only used in a minority of centers (4%) for pelvimetric measurements, with CT being the most commonly used technique (69%). This study concluded that the movement towards radiation-free techniques for pelvimetry was limited and that there were still many hospitals that had not implemented a drastic policy to reduce radiation exposure in this indication [19].

Although we could not find in the literature a recent evaluation of the situation regarding the choice of pelvic imaging techniques, the transition from radiographic or CT pelvimetry to MRI is far from being complete, with many centers still performing CT as a routine imaging modality for pelvimetry. In addition to its limited availability and longer examination time, MRI pelvimetry also suffers from the limited contrast between bone and soft tissues in “conventional” MRI sequences for pelvimetric measurements [13–18, 34–37]. In contrast, MRI-based pseudo-CT sequences, with their short echo time sensitization, optimize the visualization of mineral bone and improve its differentiation from surrounding soft tissues. These sequences have short acquisition times and have been shown to be useful as an adjunct to “conventional” MRI sequences in many rheumatological, orthopedic, or oncological indications [38]. In the current study, these sequences are used to measure obstetrical diameters, as is routinely done with CT. The added value of BB and ZTE compared to standard MRI sequences lies in their enhancement of the distinction of mineralized bone structures from soft tissues, potentially improving measurement accuracy and bridging the gap between MRI and CT for pelvimetric assessment.

Our results show that repeatability (*i.e*., the precision with which a given reader repeats the same measurement on the same subject using the same technique) is not dependent on either the imaging technique or the pelvic diameter. It does, however, depend on the reader. For reader R3, the repeatability range was similar in CT (from ± 0.26 cm to ± 0.27 cm), ZTE (from ± 0.29 cm to ± 0.33 cm), and BB (from ± 0.30 cm to ± 0.46 cm); all these values are below the “clinically acceptable” deviation of ± 0.50cm reported by Korhonen et al [34]. For the less experienced reader (Reader 4), all repeatability values were slightly higher, in CT (from ± 0.40 cm to ± 0.48 cm), in ZTE (from ± 0.44 cm to ± 0.67 cm, and in BB (from ± 0.47 cm to ± 0.52 cm). Nevertheless, these repeatability values using MRI were similar to those reported by Korhonen et al [34], where the intra-reader standard deviation ranged from 0.13 cm to 0.37 cm depending on the diameter (corresponding to a repeatability coefficient of 1.96 * standard deviation at a 95% confidence level, *i.e*., from ± 0.25 cm to ±0.73 cm). These differences in repeatability may be related to training quality and reader experience with pseudo-CT sequences.

Our results show that the reproducibility (*i.e*., the precision with which different readers repeat the same measurement on the same subject using the same technique) depends on the measured pelvic diameter. This was also reported by Korhonen et al [34], where the inter-reader standard deviation ranged from 0.16 cm to 0.60 cm depending on the diameter (corresponding to a reproducibility coefficient at a 95% confidence level of ± 0.31 cm to ± 1.18 cm). In our study, CT, ZTE, and BB have similar reproducibility in measuring OCD (± 0.39, ± 0.41, and ± 0.42 cm, respectively) and in measuring MTD (± 0.53, ± 0.53, and ± 0.58 cm, respectively). However, when examining the overlap of the 95%CIs, it was observed that while the reproducibility of ISD measurements was not statistically different in CT (± 0.26 cm (0.18 cm; 0.35 cm)) compared to ZTE (± 0.42 cm (0.29 cm; 0.55 cm)), this reproducibility was slightly different in CT compared to BB (± 0.53 cm (0.36 cm; 0.69 cm)). It is also worth noting that the reproducibility of OCD and ISD measures (either using CT or ZTE) was below the 0.5 cm deviation, while the reproducibility of MTD measures (either using CT, ZTE, or BB) slightly exceeds this deviation.

While most published series have set the clinically acceptable margin of error at ± 0.5 cm [14, 17, 34, 35], the same studies agree on the fact that reproducibility may be more difficult to achieve for certain pelvic diameters. These differences in reproducibility, also observed in our study, suggest that patient-specific factors may influence image quality and measurement accuracy, suggesting, in return, the need for standardized training protocols in pelvimetric measurement. In the current study, in which an older patient population was studied, some “anatomical” features may partly explain the variability in measurements: the presence of osteophytes affecting the anterosuperior angle of the first sacral vertebra and deformities related to previous fractures may have complicated the choice of landmarks for taking some pelvic diameter measurements.

We found a single study that evaluated the inter-technique agreement between CT and MRI, using a “conventional” T2-weighted FSE sequence for MRI pelvimetry [39]. According to the width of the agreement interval on the reported Bland-Altman plots (divided by 2 to obtain an agreement coefficient at the 95% confidence level), this study observed inter-technique agreement values ranging from ± 0.42 cm to ± 0.98 cm for the different obstetric diameters. Differences in patient positioning were proposed as the main cause of discrepancies in pelvic diameter measurements between CT and MRI. Our results show a similar level of agreement between CT and MRI, regardless of the MRI pseudo-CT sequence.

This observation holds for “All readers” (*i.e*., taking into account inter-reader variability) as well as for individual readers (*i.e*., considering intra-reader variability only). The inter-technique agreement in “All readers” ranged from ± 0.57 cm to ± 0.78 cm for CT *versus* BB, and from ± 0.63 cm to ± 0.81 cm for CT *versus* ZTE, above the reported “acceptable” deviation. When the reader was experienced (Reader 3 had longer training compared to Reader 4 in the current study) in reading pseudo-CT sequences, inter-technique agreement was good regardless of MRI sequence and diameter, with a worst value of ± 0.47 cm below the clinically acceptable deviation, suggesting that CT and MRI can be interchanged under these conditions.

Of note, either in “All readers” or in individual readers, no systematic over- or under-estimation in the diameter measurements by either of the techniques was observed, except for a marginal overestimation of + 0.16 cm with ZTE compared to CT in measuring ISD.

This study has several limitations. First, the small sample size of 21 patients limits the generalizability of our findings. Second, the images were obtained from a previous oncology study with an older population compared to the pregnant women population, as we lacked a younger cohort of patients to directly compare MRI pseudo-CT sequences with CT. Age-related anatomical changes, such as previous fractures or osteophytes, may have influenced the measurement accuracy and reproducibility. In addition, we limited our analysis to three diameters commonly requested by obstetricians, although the 3D capabilities of both ZTE and BB allow for further diametric measurements, as previously shown in 3D CT and MRI studies [31, 39]. Other pregnancy-specific observations, such as hypothetical motion artefacts due to fetal movement, remain to be evaluated in a population of pregnant patients.

In terms of examination duration, each pseudo-CT sequence took less than 3 min using the current protocol. These sequences can be further optimized for use in pregnant women who may experience vena cava compression due to prolonged dorsal decubitus. Adjustments are being made in an ongoing prospective study of pregnant women referred for CT pelvimetry.

Before this implementation for pregnant women, the theoretical risk of heating effects should be considered. The issue of heating cannot be separated from a key parameter, the specific absorption rate (SAR), which refers to the power (*i.e*., energy per unit of time) absorbed by a patient during an MRI scan.

The International Electrotechnical Commission has set a SAR limit of 2 W/Kg in normal scanning mode (4 W/Kg in first level mode), which is considered safe for all patients regardless of their condition, including pregnancy As a precaution for pregnant patients, SAR is limited to 2 W/Kg [40, 41]. Of note, SAR values delivered by the pseudo-CT MRI sequences used in the current study were 0.19 W/Kg for the ZTE sequence and 0.05 W/Kg for the BB sequences. As another future direction, the potential interest of deep learning methods, such as generative adversarial network-based approaches, to improve the contrast of ZTE and BB images to more closely resemble CT and their impact on measurements and inter-rater reliability deserves further investigation.

Getzmann et al [25] recently investigated deep learning-based synthetic pseudo-CT images derived from ZTE MRI sequences for anatomical evaluation and preoperative planning by providing pelvic or hip measurements. The present study addresses a specific clinical indication, *i.e*., obstetric pelvimetry, compares the value of native ZTE and BB sequences, evaluates the reproducibility and repeatability of manual measurements of obstetric diameters by a large group of readers, and assesses the inter-observer agreement between native pseudo-CT MRI sequences and conventional CT. The information that both the ZTE and BB sequences appear to be adequate for performing pelvimetry is interesting, as some MRI magnets may only provide one of these approaches, depending on the model or manufacturer.

In conclusion, both ZTE and BB pseudo-CT MRI sequences provide repeatable obstetric diameter measurements, with an inter-technique agreement with CT within ± 0.5 cm when read by a trained reader. However, inter-reader variability can affect the reproducibility and, thus, the inter-technique agreement. This highlights the need for adequate reader training. We believe that these radiation-free pseudo-CT MRI sequences are a promising alternative to CT for pelvimetric measurements. This preliminary proof-of-concept study encourages their evaluation and comparison with CT for pelvimetric measurements under real conditions in pregnant women.

## Data Availability

Data generated or analyzed during the study are available from the corresponding author by request.

## Abbreviations

3D: Three-dimensional
BB: Black bone
CI: Confidence interval
ICC: Intraclass correlation coefficient
ISD: Interspinous diameter
MTD: Median transverse diameter
OCD: Obstetric conjugate diameter
PET: Positron emission tomography
SAR: Specific absorption rate
ZTE: Zero echo time
